# The Interplay Between Hypocalcemia and Atrioventricular Nodal Blocking Agents in Inducing a Bradycardia, Renal Failure, Atrioventricular Nodal Blockade, Shock, and Hyperkalemia (BRASH)-Like Phenomenon With Serial Serum Drug Concentrations

**DOI:** 10.7759/cureus.78925

**Published:** 2025-02-12

**Authors:** Fumiya Inoue, Yuji Okazaki, Toshihisa Ichiba, Takuyo Chiba, Akira Namera

**Affiliations:** 1 Emergency Medicine, Hiroshima City Hiroshima Citizens Hospital, Hiroshima, JPN; 2 Emergency Medicine, International University of Health and Welfare, Chiba, JPN; 3 Forensic Medicine, Hiroshima University, Hiroshima, JPN

**Keywords:** atrioventricular nodal-blocking agents, brash syndrome, drug concentration, electrolyte disturbances, hypocalcemia

## Abstract

Renal failure is often associated with bradycardia, atrioventricular (AV) blockade, shock, and hyperkalemia, and this syndrome is known as Bradycardia, Renal Failure, AV nodal blockade, Shock, and Hyperkalemia (BRASH), which is caused by synergistic interactions between AV nodal blocking agents and hyperkalemia. However, the role of serum concentrations of AV nodal-blocking agents in this syndrome remains unclear. Furthermore, hypocalcemia, although not traditionally associated with BRASH syndrome, may have similar hemodynamic effects, such as shock and bradycardia, when combined with AV nodal-blocking agents. We report a BRASH-like phenomenon triggered by hypocalcemia and AV nodal-blocking agents.

A 69-year-old male patient with hypertension and diabetes mellitus presented five days after the onset of watery diarrhea. On presentation, his heart rate was 48 beats per minute with atrial fibrillation, and his blood pressure was 55/33 mmHg. Initial laboratory findings showed hypokalemia (2.3 mmol/L), hypocalcemia (ionized calcium of 0.96 mmol/L), and acute kidney injury (creatinine of 4.11 mg/dL). Hypotension and bradycardia persisted despite fluid resuscitation, requiring norepinephrine and atropine administration. Serum drug concentrations on admission revealed therapeutic levels of atenolol and supratherapeutic levels of amlodipine and telmisartan, despite no overdose. Hemodynamic instability persisted even after serum drug concentrations normalized on day 1. He recovered with supportive care, including electrolyte supplementation, and was discharged without complications.

This case highlights that hypocalcemia may act as an alternative trigger for a BRASH-like phenomenon, mimicking the effects of hyperkalemia. Serial drug concentration measurements suggested a synergistic interaction between AV nodal blocking agents and hypocalcemia. Given the increasing use of these medications, awareness of BRASH-like phenomena driven by electrolyte disturbances is essential. Further studies are needed to clarify the mechanisms and optimal management strategies for these conditions.

## Introduction

Bradycardia, Renal Failure, Atrioventricular (AV) nodal blockade, Shock, and Hyperkalemia (BRASH) syndrome has recently been established as a clinical entity [1]. This syndrome results from a synergistic interaction between AV nodal-blocking agents, such as beta-blockers and calcium channel blockers, with hyperkalemia, leading to severe hypotension and bradycardia [1-3]. A vicious cycle of hypotension, bradycardia, and reduced renal perfusion results in the accumulation of AV nodal-blocking agents and elevated potassium levels, further exacerbating hemodynamic instability [1-3].

However, the contribution of AV nodal-blocking agents to the pathophysiology of BRASH syndrome remains poorly understood due to the lack of data on measured drug concentrations in affected patients [4,5]. Moreover, in patients taking AV nodal-blocking agents, hypocalcemia, which impairs myocardial contractility and cardiac conduction, may also exert negative synergistic effects similar to those observed in BRASH syndrome despite the absence of hyperkalemia. This raises the possibility of a hypocalcemia-driven BRASH-like phenomenon, although this condition has not been well characterized.

We present a case of bradycardia, renal failure, AV blockade, and shock in association with hypocalcemia. Serial measurements revealed supra-therapeutic serum concentrations of AV nodal-blocking agents, suggesting a clinical picture similar to BRASH syndrome with a slightly different cause and pathophysiology. This case also highlights the possibility that mild hypocalcemia could trigger a BRASH-like phenomenon.

## Case presentation

**Figure 1:**
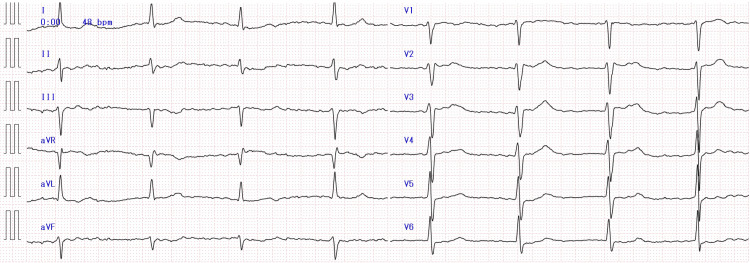
12-lead ECG at admission The initial ECG revealed atrial fibrillation with a heart rate of 48 beats per minute ECG: electrocardiogram

A 69-year-old male patient (height 167 cm, weight 97 kg, body mass index 34.8 kg/m^2^, and body surface area 2.11 m^2^) with hypertension and diabetes mellitus, who was taking 75 mg of atenolol, 10 mg of amlodipine, 80 mg of telmisartan, 500 mg of metformin, and 10 mg of empagliflozin, daily presented with watery diarrhea lasting five days. He had not taken precipitating drugs for five days due to diarrhea, but he had taken the drugs on the admission day. He had no history of chronic kidney disease (CKD) and heart disease. On arrival, his vital signs were as follows: body temperature of 35.3°C, heart rate of 48 beats per minute with atrial fibrillation, blood pressure of 55/33 mmHg, and oxygen saturation of 97% on room air. The initial electrocardiogram (ECG) revealed atrial fibrillation with slow ventricular response (Figure 1).

Laboratory examinations showed severe acute kidney injury, hyponatremia, hypokalemia, and hypocalcemia (Table 1).

**Table 1:** Laboratory examinations on admission ED: emergency department; ALT: alanine transaminase; AST: aspartate transferase; γ-GTP: γ-glutamyl transpeptidase; ALP: alkaline phosphatase; LDH: lactate dehydrogenase; ACTH: adrenocorticotropic hormone; TSH: thyroid stimulating hormone; FT4: free thyroxine

Parameters	ED admission	Reference range
White blood cell counts	8,900	3.3-8.6 × 10^3^/μL
Hemoglobin	15.7	13.7-16.8 g/dL
Platelet	21.9	15.8-34.8 × 10^4^/μL
AST	27	13-30 U/L
ALT	49	10-42 U/L
γ-GTP	30	13-64 U/L
ALP	45	38-113 U/L
LDH	262	124-222 U/L
Blood urea nitrogen	82	8-20 mg/dL
Creatinine	4.11	0.65-1.07 mg/dL
Creatinine kinase	399	59-248 U/L
C-reactive protein	3.52	<0.14 mg/dL
Procalcitonin	0.79	<0.5 ng/mL
Sodium	111.7	138-145 mmol/L
Potassium	2.3	3.6-4.8 mmol/L
Corrected calcium	8.6	8.8-10.1 mg/dL
Magnesium	2.2	1.9-2.5 mg/dL
Ionized calcium	0.96	1.08-1.3 mmol/L
Venous lactic acid	3.6	0.5-1.6 mmol/L
Cortisol	36.1	7.07-19.6 μg/dL
ACTH	20.5	7.2-63.3 pg/mL
TSH	1.31	0.61-4.23 mIU/L
FT4	1.4	0.9-1.7 ng/dL
Serum osmolarity	278	275-290 mOsm/L

**Figure 2:**
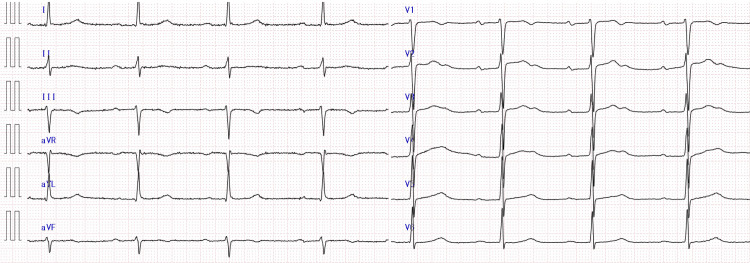
12-lead ECG on day 1 The ECG on day 1 revealed a heart rate of 48 beats per minute with sinus rhythm and first-degree atrioventricular block ECG: electrocardiogram

Cardiac ultrasonography revealed a left ventricular ejection fraction greater than 50% by visual estimation, an inferior vena cava diameter of 6 mm, no left ventricular hypertrophy, and no left atrial enlargement. Hypotension and bradycardia continued despite a bolus infusion of 1,000 mL crystalloids and 0.5 mg atropine. Thus, we administered a continuous intravenous infusion of 2 mL/kg/hour balanced fluid and 0.2 µg/kg/minute norepinephrine. We admitted him to the intensive care unit (ICU) with electrolyte supplementation without antibiotics. On day 1, his bradycardia persisted, and his ECG revealed a heart rate of 48 beats per minute with sinus rhythm and first-degree AV block (Figure 2).

**Figure 3:**
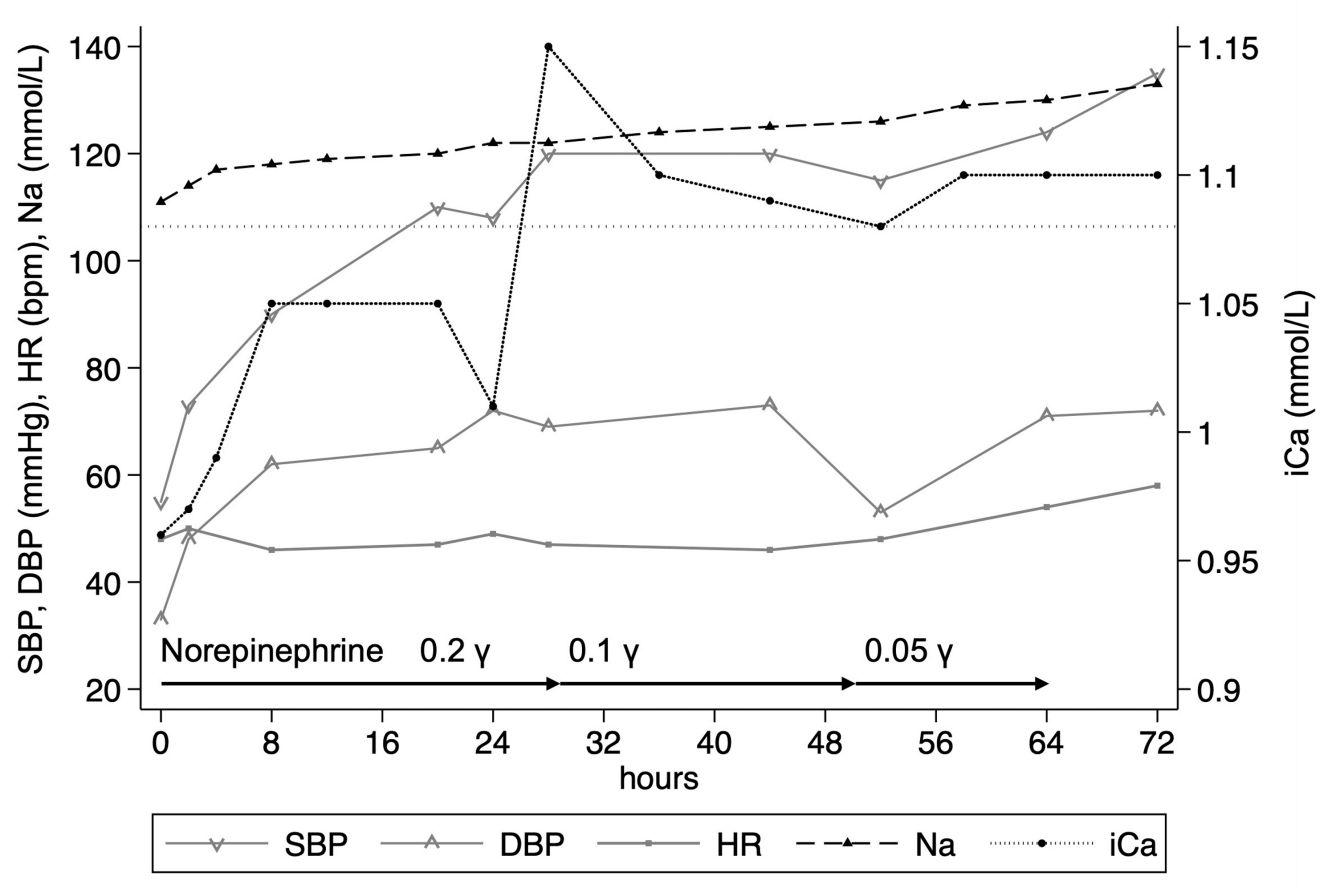
Clinical course during the ICU stay SBP: systolic blood pressure; DBP: diastolic blood pressure; HR: heart rate; Na: sodium; iCa: ionized calcium; ICU: intensive care unit

This was a case of hypovolemic hyponatremia (fluid disturbance) associated with mild lactic acidosis (acid-base disturbance) caused by lower gastrointestinal syndrome. Thus, we administered approximately 46 mL/kg (4,500 mL) of balanced fluids for the first 24 hours after ICU admission. The norepinephrine infusion was reduced to 0.1 µg/kg/minute, and the urine output increased to 1 mL/kg/hour. Laboratory data showed a potassium level of 2.8 mmol/L, corrected calcium of 9.0 mg/dL, ionized calcium (iCa) of 1.05 mmol/L, and lactic acid of 1.4 mmol/L. On day 3, the norepinephrine infusion was discontinued, the first-degree AV block was resolved, and the heart rate recovered to 56 beats per minute with sinus rhythm. Total fluid administration was approximately 113 mL/kg (11,000 mL) for 72 hours after ICU admission (Figure 3).

By day 13, his sodium level was 140 mmol/L, his potassium level was 3.6 mmol/L, and the corrected calcium level was 9.9 mg/dL. His estimated glomerular filtration rate and creatinine had improved to 34 mL/minute/1.73 m² and 1.64 mg/dL, respectively. Blood and stool cultures were negative. He was discharged without complications. Based on his clinical course, we diagnosed diarrhea as a viral infection.

A high-performance liquid chromatograph/tandem mass spectrometer (1260 infinity LC system and 6420 Triple Quad Mass spectrometer, Agilent Technologies, Palo Alto, CA) revealed that serum concentrations of atenolol were 0.7 mg/L at admission and 0.41 mg/L at 29 hours after admission (therapeutic level: 0.1-1.0), amlodipine concentrations were 0.019 and 0.007 mg/L (therapeutic level: 0.003-0.015), and telmisartan concentrations were 0.59 and 0.11 mg/L (therapeutic level: 0.006-0.225) [6]. In addition, the serum concentration of metformin was 1.8 μg/mL (therapeutic level: 0.1-2) at admission.

## Discussion

**Figure 4:**
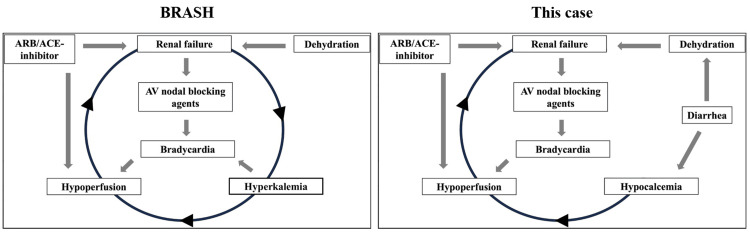
Clinical pictures of BRASH syndrome and BRASH-like phenomenon induced by hypocalcemia and AV nodal blocking agents AV: atrioventricular; ARB: angiotensin receptor blocker; ACE: angiotensin-converting enzyme; BRASH: Bradycardia, Renal Failure, AV nodal blockade, Shock, and Hyperkalemia

This case highlights the importance of evaluating serial serum concentrations of AV nodal blocking agents in conjunction with electrolyte abnormalities in patients presenting with bradycardia, renal failure, AV nodal blockade, and shock, which have clinical features similar to those of BRASH syndrome. Notably, this condition developed in the presence of hypocalcemia rather than hyperkalemia, suggesting that hypocalcemia may act as an alternative trigger for hemodynamic instability when combined with AV nodal-blocking agents (Figure 4).

The BRASH syndrome has been described as a negative synergy between AV nodal blocking agents and hyperkalemia, which is considered to be distinct from that of isolated intoxication with these drugs or isolated hyperkalemia [2]. However, the exact mechanisms underlying this synergistic effect remain unclear. In other words, it is not fully understood whether it is caused by excessive sensitivity to drugs at therapeutic serum concentrations or by elevated serum concentrations secondary to renal failure [1]. Data on serial measurement of serum concentrations of AV nodal blocking agents and additional agents such as angiotensin-converting enzyme inhibitors and angiotensin receptor blockers during the development of BRASH syndrome are limited. Previous reports include only two cases with single-point measurements [4,5]. One case involved a therapeutic concentration of metoprolol precipitating symptoms [4], while another involved amlodipine overdose, with a serum concentration twice the therapeutic range [5]. In our case, serial measurements revealed that atenolol was within the therapeutic range on admission, amlodipine was slightly supratherapeutic, and telmisartan was twice the therapeutic range. By day 1, serum concentrations of all drugs returned to the therapeutic ranges despite the patient requiring norepinephrine infusion at 0.1 µg/kg/minute to maintain hemodynamic stability. These findings suggest two implications: 1) supratherapeutic concentrations of amlodipine and telmisartan likely amplified initial severe hypotension because these serum concentrations did not reach toxic levels and 2) persistent hypotension requiring norepinephrine support, even after serum concentrations of all drugs normalized, cannot be fully explained by drug toxicity alone.

Even mild hypocalcemia may act as a trigger for hemodynamic instability when combined with AV nodal-blocking agents. Hypocalcemia can impair myocardial contractility and cardiac conduction, particularly in cases of severe hypocalcemia or underlying structural heart disease [7,8]. The cardiovascular effects of hypocalcemia, including both systolic mechanical dysfunction and electrophysiological abnormalities, are typically reversible [9]. In such cases, the median iCa level has been reported as 0.66 mmol/L [9]. Although the mild hypocalcemia observed in our case alone is unlikely to cause shock, its combination with AV nodal blocking agents may unexpectedly compromise hemodynamic stability. This phenomenon may mirror the observation that BRASH syndrome can develop even with mild hyperkalemia [2,10]. Although dehydration due to diarrhea may have contributed to the circulatory effects in our case, the response to adequate fluid resuscitation and electrolyte correction suggests that dehydration and hypocalcemia alone cannot fully explain the clinical presentation. The BRASH syndrome is typically triggered by hyperkalemia secondary to renal failure due to dehydration, especially in elderly patients [11,12]. However, in our case, diarrhea-induced dehydration led to acute prerenal injury, and severe electrolyte losses resulted in hypocalcemia, which, in combination with AV nodal blocking agents, likely precipitated hemodynamic compromise. This suggests that the type and severity of electrolyte disturbances, as well as the degree of dehydration, may influence whether BRASH syndrome manifests as hyperkalemia-driven or hypocalcemia-driven variants.

In addition, the unique phenomena observed in our case may be attributable to the effects of CKD on myocardial conduction [13]. CKD is associated with various cardiovascular abnormalities, including arrhythmias and conduction disturbances. In the early stages of CKD, subtle electrolyte imbalances may occur due to the kidneys’ impaired ability to reabsorb ions such as potassium and sodium. These imbalances alter the membrane potential of cardiac myocytes, making them more prone to arrhythmias. As CKD progresses, impaired renal filtration and excretory function exacerbate these electrolyte abnormalities, often resulting in conditions such as hyperkalemia and fluid overload. These changes can severely affect the heart’s electrical conduction system, causing conduction blocks and various arrhythmias. Moreover, the accumulation of uremic toxins and the chronic inflammatory state associated with CKD may further contribute to the structural and electrical remodeling of the myocardium due to cardiac fibrosis [14]. Thus, the progression of CKD may potentially play a role in the development of cardiac rhythm and conduction abnormalities. In our case, in addition to hypocalcemia and AV nodal blocking agents, the effects of renal impairment may also have contributed.

## Conclusions

AV nodal-blocking agents and antihypertensive drugs are commonly used for treating cardiovascular diseases in the elderly. However, BRASH syndrome remains underrecognized and is likely to become more prevalent in the future. Currently, data on serum drug concentrations of these agents during BRASH syndrome and BRASH-like phenomena are scarce. Accumulating cases with detailed documentation of drug concentrations and electrolyte disturbances is essential to refine management strategies, including “sick day cessation” protocols and switching to alternative therapies. Such efforts are critical to optimizing care for BRASH syndrome and its variants.
